# CAN DETERIORATION IN BALANCE BE EASILY DETECTED IN MIDDLE AGE?

**DOI:** 10.1093/geroni/igae098.0240

**Published:** 2024-12-31

**Authors:** Shmuel springer, Roee Hayek, Silvi Frenkel Toledo, Guy Baranes

**Affiliations:** Ariel University, Ariel, HaMerkaz, Israel; Ariel University, Ariel, HaMerkaz, Israel; Ariel University, Ariel, HaMerkaz, Israel; Ariel University, Ariel, HaMerkaz, Israel

## Abstract

Balance and physical performance tend to start declining in middle-age. However, most existing tests are generally not sensitive enough to detect subtle balance disorders in this population. We aimed to evaluate the utility of the Brief Balance Evaluation Systems Test (Brief-BESTest) and accelerometry data to identify middle-aged individuals with excessive balance deterioration. Young adults (YA, 25.3±2.3 years), early middle-aged (EMA, 47.7±2.6 years), and late middle-aged (LMA, 60.6±3.6 years) individuals, 25 subjects in each group, were tested using the Brief-BESTest with accelerometer (Delsys, USA) placed over their lower-back to measure the trajectory of the center-of-mass during standing tasks (95% confidence ellipse of trajectory, 95%CE). Significant differences in Brief-BESTest scores were found between the young and the two middle-aged cohorts (P < 0.05) and between EMA and LMA (p < 0.05). The 95%CE showed significant differences between YA and LMA in all standing conditions (P < 0.05) and between EMA and LMA (p < 0.05) when standing on a foam with eyes closed. Both, the Brief-BESTest scores and the 95%CE values demonstrated increased variation with age, allowing to identify subjects in the lower interquartile range (LIQR) defined as having accelerated balance deterioration. Significant moderate correlation (R2=0.44, P=0.001) was found between Brief-BESTest and 95%CE of single-leg-stance, and 46% of individuals in the LIQR were identified by both tests. These results enhance the utility of balance testing in midlife with the long-term goal of minimizing age-related functional decline. In addition, the results suggest that balance assessment using accelerometry motion data, available on any smartphone, can further facilitate early detection of balance decline.

